# Astrocyte gap junctions and K_*ir*_ channels contribute to K^+^ buffering and regulate neuronal excitability

**DOI:** 10.3389/fncel.2025.1571218

**Published:** 2025-11-20

**Authors:** Danica Bojovic, Andre Dagostin, Steve J. Sullivan, Ben Emery, Henrique von Gersdorff, Anusha Mishra

**Affiliations:** 1 Vollum Institute, Oregon Health & Science University, Portland, OR, United States; 2 Department of Neurology, Jungers Center for Neurosciences Research, Oregon Health & Science University, Portland, OR, United States; 3 Department of Anesthesiology and Perioperative Medicine, Oregon Health & Science University, Portland, OR, United States

**Keywords:** K^+^ buffering, astrocytes, neuronal excitability, gap junctions, K_*ir*_ channels, MFA, Ba^2+^

## Abstract

Astrocytes are connected in a functional syncytium via gap junctions, which contribute to the maintenance of extracellular K^+^ homeostasis. The prevailing hypothesis is that K^+^ released during neuronal firing is taken up by astrocytes via K_*ir*_ channels and then distributed among neighboring astrocytes via gap junctions. Here, we tested the effect of blocking gap junctions and K_*ir*_ channels, both independently and simultaneously, on field excitability of cortical slices in response to a stimulation train. Independently blocking either gap junctions or K_*ir*_ channels increased the amplitude of the first fEPSC (field excitatory post-synaptic current) response, followed by suppression of both fiber volley (pre-synaptic action potentials) and fEPSCs during sustained stimulation. Surprisingly, simultaneous block of both gap junctions and K_*ir*_ channels enhanced the suppression of neuronal activity, resulting in a ∼75% decrease in fiber volley amplitude in the first response, followed by a fast and strong suppression of fEPSCs during sustained stimulation. Genetic depletion of astrocyte gap junctions showed a reduction but not complete loss of Cx43, indicating partial syncytial decoupling, and, accordingly, had a weaker but similar effect on neuronal excitability as blocking gap junctions. Pharmacological K_*ir*_ block in mice with reduced gap junction coupling suppressed sustained firing of the fiber volley but not fEPSCs. That this effect was milder than K_*ir*_ block alone suggests that adaptive mechanisms may be recruited upon genetically induced astrocyte decoupling. We conclude that K^+^ buffering via K_*ir*_ and gap junctions in astrocytes together play a critical role in maintaining neuronal excitability, particularly during sustained activity, but that other mechanisms can be recruited to perform this function in their absence.

## 1 Introduction

Neuronal excitability relies on the maintenance of ionic gradients across cell membranes. In this context, potassium ion (K^+^) gradient is characterized by high intracellular and low extracellular concentrations. Although K^+^ homeostasis is necessary for optimal neuronal firing, how extracellular K^+^ concentration ([K^+^]_*e*_) is regulated is not completely understood. Presumably, [K^+^]_*e*_ is largely regulated by astrocytes, which express high levels of inwardly rectifying K^+^ 4.1 channels (K_*ir*_4.1) that take up the K^+^ released from active neurons (Djukic et al., 2007). Astrocytes also form a syncytium connected via gap junctions (GJs), composed of connexin 30 and 43 (Cx30 and Cx43) (Hösli et al., 2022). The prevailing hypothesis is that astrocytes take up K^+^ via K_*ir*_4.1 channels and then spread it throughout the syncytium via GJs to maintain [K^+^]_*e*_ homeostasis during bouts of neuronal activity (Djukic et al., 2007; Wallraff et al., 2006).

Changes in [K^+^]_*e*_ are observed during aging and neurodegeneration (Ding et al., 2024), underscoring the importance of understanding the effect of K^+^ homeostasis on neuronal health. Studies have reported that an increase in [K^+^]_*e*_ leads to either depression (Contreras et al., 2021; Meeks and Mennerick, 2004) or hyperexcitation (Bataveljic et al., 2024; Traynelis and Dingledine, 1988) of neuronal firing, but the reason for these disparate reports are unclear. As K_*ir*_4.1 channels and GJs are thought to be the key players in buffering [K^+^]_*e*_, many studies have investigated their roles in K^+^-dependent changes of neuronal excitability. While it is established that K_*ir*_4.1 reduction results in hyperexcitability (Bataveljic et al., 2024; Inyushin et al., 2010), the contribution of GJs has been contested (Breithausen et al., 2020; Wallraff et al., 2006). This is partially due to difficulties in effectively decoupling the syncytium with available tools; pharmacological blockers are non-specific to astrocytes, while astrocyte-specific genetic knockout models sometimes do not achieve complete decoupling (Hösli et al., 2022) and can induce widespread compensatory or adaptive transcriptional changes (Djukic et al., 2007; Hösli et al., 2022). Therefore, tool limitations are a likely cause of the variability in findings of GJ contribution to K^+^ buffering. To test this notion, we used both pharmacological and genetic tools to reduce astrocyte GJs and evaluate the contribution to neuronal excitability, defined here as network activity measured by field recordings.

In response to a moderate 3 s, 20 Hz stimulation, blocking either GJs or K_*ir*_ independently resulted in similar short-lived hyperexcitability, characterized by an initial increase in post-synaptic current amplitude, followed by a fast suppression of the amplitude of both fiber volley and post-synaptic responses. Further, we found that combined block of GJs and K_*ir*_ resulted in pronounced suppression of neuronal activity: a strong reduction of both fiber volley and post-synaptic activity. We next used a Cx30KO:Cx43KD mouse model, which had a complete reduction of Cx30 but only a 40% reduction in Cx43, and accordingly observed a weaker effect on excitability. Combining Cx30KO:Cx43KD with K_*ir*_ block still produced a mild effect on excitability, showing inability to sustain fiber volley amplitude, but not affecting fEPSCs. Our data indicate that K_*ir*_ channels and GJs, likely in astrocytes, orchestrate K^+^ buffering and preserve sustained neuronal firing. However, this effect on global excitability is observed only with sufficient block of GJs and K_*ir*_ channels and is difficult to reproduce in genetic knockout model when complete ablation of GJs is not achieved.

## 2 Materials and methods

### 2.1 Animals

All experimental procedures were performed in accordance with the Institutional Animal Care and Use Committee (IACUC) at Oregon Health & Science University. C57BL/6J mice of both sexes at 4–8 weeks of age were used. A total of 35 WT mice, 12 Cx30KO:Cx43KD mice and 7 littermate controls were used in this study.

### 2.2 Genetic mouse model

Mice with global knockout of *Gjb6* gene (Cx30^–/–^) were kindly donated by Dr. Christian Steinhauser (University of Bonn, Germany). Mice with floxed-*Gja1* gene (Cx43*^fl/fl^*; Strain #008039, The Jackson Laboratories, USA) and the *^fl–stop^*tdTomato reporter were shared with us by Dr. Kevin Wright, OHSU. The two lines were bred for several generations to obtain two genotypes: Cx30^–/–^:Cx43*^fl/fl^* and Cx30^+/–^:Cx43*^fl/fl^*. To knockdown Cx43 specifically in astrocytes, AAV9-GfaABC_1_D-Cre (2.9×10^10^ GC/g in 1.5-3uL volume) was injected unilaterally into the lateral ventricle (intracerebroventricular- ICV injection) of P5-P7 Cx30^–/–^:Cx43*^fl/fl^* mice to prevent syncytial formation (Supplementary Figure 5). These mice had reduced astrocyte GJs and are referred to as Cx30KO:Cx43KD throughout the manuscript. Littermates with Cx30^+/–^:Cx43*^fl/fl^* genotype were injected with a control AAV9-GfaABC_1_D-tdTomato virus (2.9×10^10^ GC/g) and are referred to as littermate controls (although note that these mice are heterozygous for Cx30). Acute brain slices were assessed for the % area covered by tdTomato-positive astrocytes to evaluate viral recombination efficiency and analyzed in ImageJ. Reduction in Cx30 and Cx43 protein levels were evaluated using Section “2.6 Immunofluorescence” and Section “2.7 Western blot,” respectively.

### 2.3 Preparation of acute cortical slices

Cortical slices were prepared as described previously (Bojovic et al., 2022). Mice were quickly euthanized under anesthesia (4% isoflurane) and the brains removed. Cortical slices (300 μm) were cut with a Compresstome VF-300-0Z (Precisionary Instruments Inc., USA) in oxygenated (95% O_2_/5% CO_2_) ice-cold slicing solution containing 93 mM N-methyl-D-glucamine (NMDG)-Cl, 2.5 mM KCl, 0.5 mM CaCl_2_, 10 mM MgCl_2_, 1.2 mM NaH_2_PO_4_, 30 mM NaHCO_3_, 20 mM HEPES, 25 mM D-GLUCOSE, 1 mM kynurenic acid, 5 mM Na ascorbate, 3 mM Na pyruvate, 2 mM thiourea (NMGD replaces Na^+^ in the slicing solution to provide charge and osmotic pressure but prevent excitotoxicity). Brain slices were placed in a warm recovery bath with the same slicing solution at 37 °C for 20 min, then transferred to an oxygenated (95% O_2_/5% CO_2_) storage solution containing 92 mM NaCl, 2.5 mM KCl, 2 mM CaCl_2_, 1 mM MgCl_2_, 1.2 mM NaH_2_PO_4_, 30 mM NaHCO_3_, 20 mM HEPES, 25 mM D-GLUCOSE, 1 mM kynurenic acid, 5 mM Na ascorbate, 3 mM Na pyruvate, 2 mM thiourea at room temperature and allowed to equilibrate for at least 30 min before use. Experiments were performed in 1–3 slices from each animal, up to 5–6 h after slicing.

### 2.4 Electrophysiology

Slices were transferred to a recording chamber and continuously perfused (3–4 ml/min) with oxygenated (20% O_2_, 5% CO_2_, 75% N_2_) artificial cerebrospinal fluid (aCSF) composed of 124 mM NaCl, 3.5 mM KCl, 2 mM CaCl_2_, 1 mM MgCl_2_, 1 mM NaH_2_PO_4_, 26 mM NaHCO_3_, 10 mM glucose, and 1 mM Na ascorbate. Slices were visualized under an upright Olympus BX51WI microscope (Japan) under DIC optics. Borosilicate glass pipettes (World Precision Instruments, USA) were pulled into glass electrodes with a Micropipette Puller (Sutter Instruments model P-87, USA). The recording electrode resistance was ∼3–5 MΩ, while the stimulating electrode had a large diameter (15–25 μm) tip with low resistance. Both electrodes were filled with aCSF and inserted into the region of interest, with the stimulating electrode in layers I-II and recording electrode in layers IV-V of the cortex. Field currents were recorded in voltage-clamp mode (Axon Instruments, Molecular Devices, USA). All experiments were conducted at 33 °C–36 °C. Single stimulation pulses were delivered to find the field response before starting the experiment. A 30 min stabilization period was allowed, following which a 3 s, 20 Hz stimulation train was delivered to obtain baseline (pre-treatment) for each slice, and again after 1 h bath application of drugs of interest (post-treatment). Three traces were collected for baseline and drug conditions, with 2 min intervals between recordings, and averaged to represent each condition.

### 2.5 Pharmacology

AMPA receptors were blocked by 10 μM cyanquixaline (CNQX; Tocris Bioscience, UK); voltage-gated Na^+^ channels with 1 μM tetrodotoxin (TTX; Tocris Bioscience, UK); gap junctions with 100 μM meclofenamic acid (MFA; Sigma Aldrich, USA) and 100 μM carbenoxolone (CBX; Tocris Bioscience, UK); and K_*ir*_ channels with 200 μM BaCl_2_ (Sigma Aldrich, USA).

### 2.6 Immunofluorescence

Mice were anesthetized and transcardiac perfusions performed first with saline followed by 4% paraformaldehyde (PFA). The brains were removed, post-fixed in PFA overnight, then submerged in 30% sucrose gradient to dehydrate the tissue. Brains were embedded in Optimal Cutting Temperature (OCT) compound (Tissue-Tek, Sakura, USA), cryosectioned at 16 μm thickness, and stored at −80 °C. At the time of immunolabeling, sections were air dried for at least 2 h before being rehydrated in phosphate buffered saline (PBS) and washed 3× in PBS. Sections were blocked for 1 h at room temperature with 10% donkey serum (Sigma-Aldrich, USA) and 1% Bovine Serum Albumin (BSA, Vector laboratories, USA) with 0.05% Triton X-100 (Thermo Fischer Scientific, USA) in PBS. Rabbit polyclonal Cx30 antibody (1:250, Cat. no. 71-2200, Invitrogen, USA) was applied in PBS with 1% donkey serum and 0.1 % BSA and incubated overnight in a sealed humidified container at room temperature. Sections were then washed 3× in PBS and Alexa Fluor 647-conjugated donkey-anti-rabbit secondary antibody (1:1000; Cat. no. ab150075, Abcam, USA) was applied for 2 h at room temperature. Sections were washed 3× with PBS, and cover slipped with Fluoromount G (Invitrogen, USA). Images were acquired on a ZEISS LSM 980 with Airyscan 2. Cx30 positive puncta were analyzed and counted with IMARIS. The threshold for counting puncta as Cx30-positive was determined based on non-specific background in sections with only secondary antibody in Cx30^–/–^ sections.

### 2.7 Western blot

Mice were euthanized under anesthesia, brains removed, and cortical tissues dissected and frozen at −80 °C. At the time of experiment, cortical tissue was lysed by douncing in RIPA buffer (50 mM Tris-HCL pH 8.0, 150 mM NaCl, 1% NP-40, 0.5% Sodium deoxycholate, 0.1% SDS, 1 mM EDTA, 0.5 mM EGTA) with complete protease inhibitors (11873580001, Roche, Switzerland), and phosphatase inhibitors (04906837001, Roche, Switzerland) followed by spinning at 13,000 × *g* at 4 °C for 15 min. The supernatant (lysate) was removed and run on a 4%–12% Bis-Tris-gel (NP0335BOX, Invitrogen, USA) at 150 V for ∼1 h. Proteins were then transferred to a PVDF membrane (IPVH00010, Thermo Fisher Scientific, USA) at 20 V for 1 h. Following transfer, blots were rinsed in 1× TBS with 0.1% Tween-20 (TBST) before blocking in 1× TBST with 5% milk powder for 1 h at room temperature. Blots were then incubated with rabbit Cx43 antibody (1:1000, Cat. no. 3512, Cell Signaling Technologies, USA) diluted in 2.5% BSA (BP9706-100, Thermo Fisher Scientific, USA) with 1% NaN_3_ in TBST overnight at 4 °C while shaking. Blots were then washed 3× in TBST and incubated with HRP-conjugated goat anti-rabbit secondary (Cat. no. 7074, Cell Signaling) at 1:5000 with 2% milk powder in TBST for 2 h at room temperature with shaking. The blots were imaged using chemiluminescence (34080, Thermo Fisher Scientific, USA) on a Syngene GBox iChemiXT to visualize immunoreactivity. Following this, blots were relabeled with β-actin-HRP (1:5000; A3854, Sigma-Aldrich, USA) and imaged. Densitometric analysis was performed in ImageJ by quantifying the intensity of bands relative to the β-actin loading control and normalized to background.

### 2.8 Data analysis

Field recordings were analyzed in Igor Pro with Neuromatic extension (Wave Metrics, USA). Statistical analysis was performed in Prism (GraphPad, USA) and Excel (Microsoft, USA). All data are presented as median (interquartile range). The significance level was set at *p* < 0.05.

All traces were first smoothed with binomial function. Peak amplitude was calculated as the difference between peak of the fiber volley (pre-synaptic action potentials) or fEPSC (field post-synaptic current) and the average baseline value before stimulation. To calculate the fold change in amplitude after each treatment, the first post-treatment amplitude after 1 h was normalized to the first pre-treatment amplitude. To calculate the amplitude change over the 3 s, 20 Hz stimulation, each of the 60 traces were normalized to the first trace. Time to 50% amplitude was calculated by finding the time point when amplitude decreased below 50% of the maximal amplitude change over the 3 s stimulation. Data that failed any of the normality tests in GraphPad Prism were deemed non-parametric. Wilcoxon test was used to compare paired pre-treatment to post-treatment values. Mann-Whitney test was used to compare unpaired samples between two groups. Kruskal-Wallis test was used for comparison of unpaired samples between multiple groups.

## 3 Results

### 3.1 Experimental setup for assessing neuronal field activity

**FIGURE 1:**
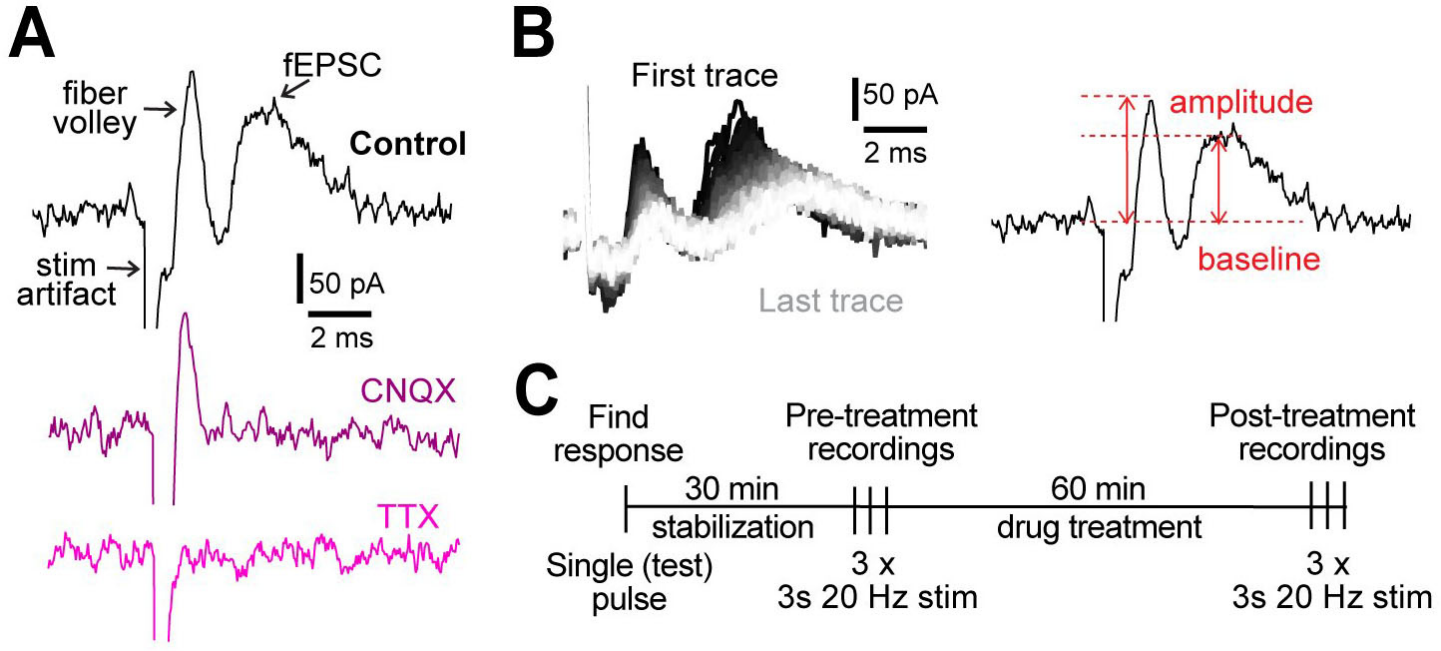
Experimental design. **(A)** Representative examples of field recordings showing that the post-synaptic currents are blocked by 10 μM CNQX (purple) and fiber volley by 1 μM TTX (pink). **(B)** A representative example of all 60 traces from a 3 s, 20 Hz stimulation (left). The order of traces is represented with a gradient, first (black) to last (white). Peak amplitude was measured as the difference between the highest point in fiber volley and fEPSC compared to the baseline (right). **(C)** Experimental protocol used for all experiments.

We measured evoked field activity in cortical layers IV/V in response to 3 s, 20 Hz stimulation as a readout of neuronal excitability. We defined neuronal excitability in terms of local network activity in the region of recording, whereby fiber volley indicates the number of stimulated presynaptic neurons and field excitatory post-synaptic currents (fEPSC; Figure 1A) indicate overall strength of postsynaptic neuronal depolarizations. Blocking AMPA receptors with 10 μM cyanquixaline (CNQX; Figure 1A) abolished the second peak, confirming it as fEPSC, while blocking voltage-gated Na^+^ channels with 1 μM tetrodotoxin (TTX; Figure 1A) abolished both fiber volley and fEPSC (but not the stimulation artifact), showing that the first peak represents the pre-synaptic fiber volley. Field recordings were allowed to stabilize for 30 min, a period during which the signal strength steadily increased (Supplementary Figure 1), likely due to the large diameter opening of the stimulation electrode sealing with the tissue. After stabilization, the 3 s, 20 Hz stimulation (Figure 1B) was delivered 3 times in 2 min intervals to record and quantify the neuronal activity and repeated after 1 h treatment under different conditions (Figure 1C).

Under control conditions (aCSF), we measured an increase in neuronal post-synaptic response to the 3 s, 20 Hz stimulation after 1 h compared to the pre-treatment. The peak amplitude of the first fiber volley stayed similar: it was 85.9 pA [61.9–103.5] (data reported as median [IQR]) at the start and 96.5 pA [67.2–113.7] after 1 h (*p* = 0.07; Supplementary Figure 2A). However, the peak amplitude of first fEPSC increased from 95.9 pA [77.6–156.9] at the start to 152.1 pA [124.6–276.1] after 1 h (*p* < 0.0001; Supplementary Figure 2D). While the 30 min stabilization period (Supplementary Figure 1) tapered the increase in fEPSC, they continued to increase throughout the 1-h recording period.

### 3.2 Gap junctions and K_*ir*_ channels independently regulate neuronal activity

**FIGURE 2:**
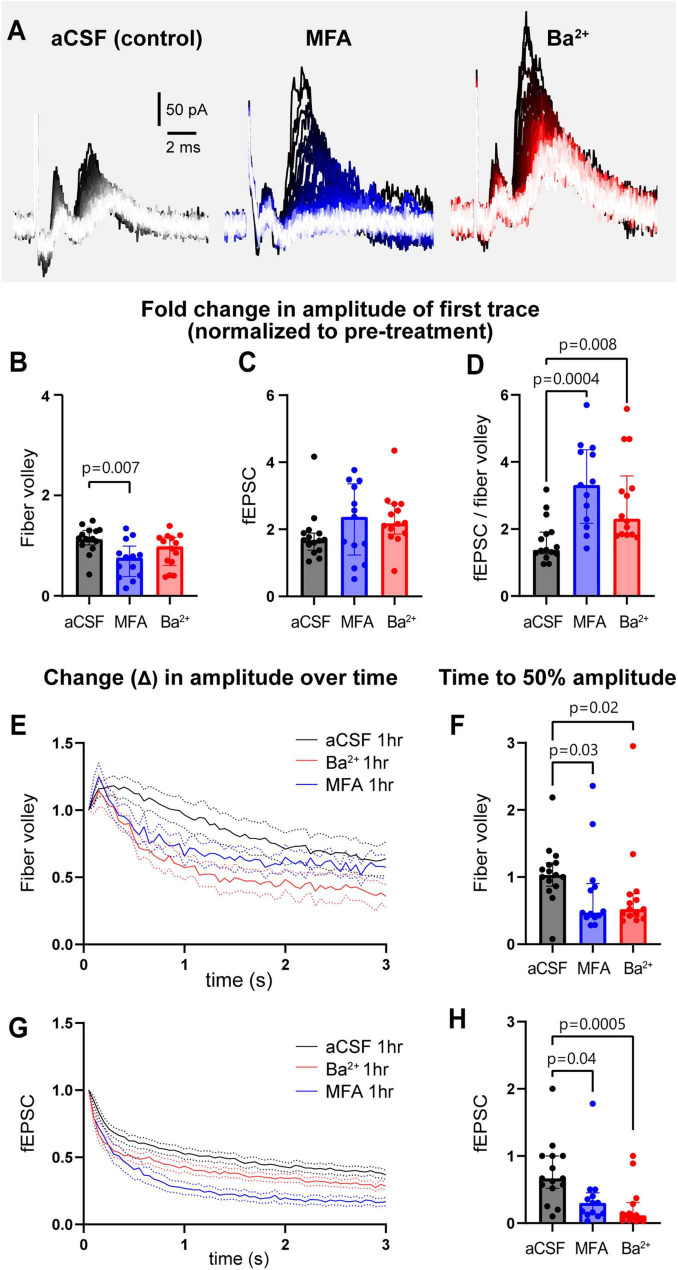
GJs and K_*ir*_ channels regulate neuronal field responses and contribute to maintenance of sustained neuronal activity. **(A)** Representative traces of recordings from slices treated with aCSF, the GJ blocker MFA, and K_*ir*_ channel blocker Ba^2+^. **(B)** The fold change in the amplitude of first fiber volley showing a relative decrease after 1 h incubation in MFA, but not Ba^2+^, compared to aCSF. **(C)** The fold change in the amplitude of the first fEPSC after 1 h in MFA and Ba^2+^ was similar to aCSF. **(D)** The fEPSC:fiber volley ratio showed a significant increase in both MFA and Ba^2+^. **(E)** The decrease in normalized fiber volley amplitude over 3 s, 20 Hz stimulation after 1 h in aCSF, MFA, and Ba^2+^. **(F)** Fold change in fiber volley time to 50% showing the relative decrease after 1 h in MFA and Ba^2+^. **(G)** The decrease in normalized fEPSC amplitude over 3 s, 20 Hz stimulation after 1 h in control, MFA, and Ba^2+^. **(H)** Fold change in fEPSC time to 50% showing the relative decrease after 1 h in MFA and Ba^2+^. aCSF control *N* = 15 mice, 15 slices; MFA *N* = 13 mice, 13 slices, Ba^2+^ *N* = 13 mice, 14 slices. Kruskal-Wallis test; data shown as median [IQR].

The 3 s, 20 Hz stimulation was repeated before and after 1 h treatment with MFA (gap junction blocker) and Ba^2+^ (K_*ir*_ channel blocker) applied in the bath (Figure 2, Supplementary Figure 2). To examine the effect of GJs on neuronal activity, we chose MFA, a GJ blocker shown to have higher potency than other commonly used blockers (Pan et al., 2007). MFA begins blocking GJ coupling between astrocytes after 15–25 min exposure, although not completely, resulting in increased input resistance and decreased capacitance of astrocytes (Schools et al., 2006). Complete block of GJs with 100 μM MFA was observed after 1 h in retinal amacrine neurons (Veruki and Hartveit, 2009), thus we used 1 h incubations to test the effect of GJ on neuronal activity in our studies. The fold change in the amplitude of the first fiber volley after 1 h of MFA treatment was smaller compared to control aCSF condition (aCSF: 1.1 [1.0–1.3]; MFA: 0.8 [0.4–1.0], *p* = 0.007; Figure 2B), while that after Ba^2+^ treatment was somewhat lower but not statistically significant (Ba^2+^: 1.0 [0.6–1.2], *p* = 0.2, Figure 2B). Surprisingly, neither MFA nor Ba^2+^ affected the fold change in the first peak fEPSC amplitude (aCSF: 1.7 [1.4–1.9]; MFA: 2.4 [1.2–3.4], *p* = 0.4; Ba^2+^: 2.2 [1.9–2.8], *p* = 0.06; Figure 2C). The decrease in fiber volley despite the fEPSCs remaining similar in size suggested that the size of the post-synaptic response per pre-synaptic axon stimulated was different between conditions. Thus, we calculated the fEPSC:fiber volley ratio for each condition. We found that the fEPSC:fiber volley ratio almost doubled in both MFA and Ba^2+^ compared to control (aCSF = 1.4 [1.3–1.9]; MFA = 3.3 [2.2–4.4], *p* = 0.0004; Ba^2+^ = 2.3 [1.8–3.6], *p* = 0.008; Figure 2D). These data show that both K_*ir*_ and GJs regulate neuronal excitability in a similar manner.

When examining the response over the course of the 3 s, 20 Hz stimulation train, we observed that the amplitude of the fiber volley and fEPSCs both steadily decreased over 3 s of stimulation (Figures 2E, G). To quantify this decrease in neuronal activity, we plotted the amplitude of each fiber volley and fEPSC over the 3 s period and quantified the time it takes for amplitude to decrease below 50% of the maximum change measured at the end of the stimulation train. Both fiber volley and fEPSC amplitudes decreased faster in presence of MFA or Ba^2+^ compared to aCSF. Fiber volley decreased 50% faster in both MFA and Ba^2+^-treated slices (fold change in time to 50% amplitude in aCSF: 1.0 [0.9–1.2]; MFA: 0.5 [0.4–0.9], *p* = 0.03; Ba^2+^: 0.5 [0.4–0.8], *p* = 0.02, Figures 2E, F). The decrease in fEPSC was more pronounced: 57% faster in MFA and 86% faster in Ba^2+^-treated slices (aCSF: 0.7 [0.5–1.0]; MFA: 0.3 [0.1–0.5], *p* = 0.04, Ba^2+^: 0.1 [0.0–0.3], *p* = 0.0005; Figures 2G, H; also see Supplementary Figure 2).

We observed that the size of the first neuronal responses increased over time even in aCSF (Supplementary Figures 1, 2). This suggests that, for studies that use field recordings, it is important to time experiments similarly when comparing different conditions so as to minimize artifactual differences. We also note the considerable variability of responses in all conditions that could not be explained by age (Supplementary Figure 3A) or sex (Supplementary Figure 3B) of the mouse.

We also examined a second inhibitor of GJs, carbenoxolone (CBX), which is more commonly used but is less potent (Pan et al., 2007 and Supplementary Figure 4). CBX (100 μM) had a surprisingly mild effect on neuronal activity: first fiber volley amplitude increased by 29 %, from 83.2 pA [64.1–113.6] pre-treatment to 107.4 pA [86.7–131.3] after 1 h in CBX (*p* = 0.008; Supplementary Figure 4A), but it had no statistical effect on fEPSC amplitude (pre-treatment: 97.6 pA [64.6–126.7]; post-treatment = 114.9 pA [78.0–199.0]; *p* = 0.08; Supplementary Figure 4B). Compared to aCSF control after 1 h, the fold change in fiber volley amplitude (aCSF: 1.1 [1.0–1.3]; CBX: 1.2 [1.1–1.5], *p* = 0.3; Supplementary Figure 4C), fEPSC amplitude (aCSF: 1.7 [1.4–1.9]; CBX: 1.2 [0.9–1.7], *p* = 0.09; Supplementary Figure 4D), and the fEPSC:fiber volley ratio (aCSF: 1.4 [1.2–1.9]; CBX: 1.1 [0.8–1.8], *p* = 0.2; Supplementary Figure 4E) were all unchanged. CBX had a weak effect on the rate of decrease of fiber volley over the 3 s, 20 Hz stimulation with time to 50% decreasing from 2 s [1.3–2.2] in pre-treatment to 1.0 s [0.7–1.2] after 1 h in CBX (*p* = 0.02; Supplementary Figures 4F, G). There was no statistical effect of CBX on the time to 50% of fEPSC (pre-treatment: 1.3 s [0.3–2.3]; post-treatment: 0.8 s [0.5–0.9]; *p* = 0.3; Supplementary Figures 4H, I).

### 3.3 Gap junctions and K_*ir*_ channels coordinate together to regulate neuronal activity

**FIGURE 3:**
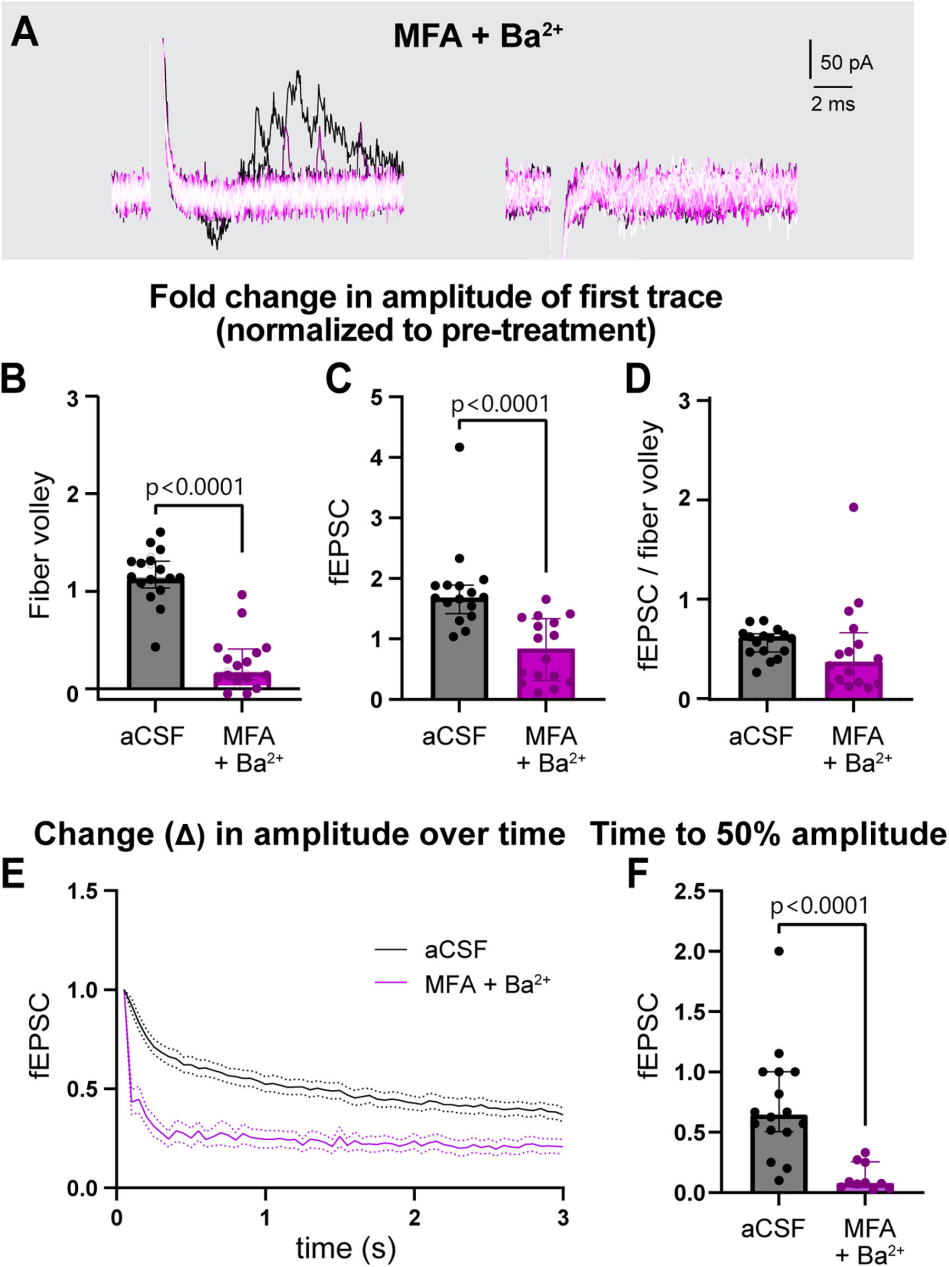
Simultaneous treatment with MFA and Ba^2+^ strongly disrupted neuronal activity. **(A)** Representative traces of field recording during 3 s, 20 Hz stimulation in presence of MFA + Ba^2+^. **(B,C)** Fold change in fiber volley **(B)** and fEPSCs **(C)** amplitudes were smaller in MFA + Ba^2+^ compared to aCSF controls. **(D)** Fold change in fEPSC:fiber volley ratio showed no change. **(E)** Average decrease in normalized fEPSC amplitude during the 3 s, 20 Hz stimulation after 1 h in MFA + Ba^2+^ and aCSF. **(F)** Time to 50% fEPSC amplitude was smaller compared to aCSF control. aCSF control data are the same as in Figure 2. aCSF *N* = 15 mice,15 slices; MFA + Ba^2+^ *N* = 11 mice, 16 slices. Mann-Whitney test; data shown as median [IQR].

The similar effect of blocking GJs and K_*ir*_ channels independently suggests that they may work together to regulate neuronal activity. To examine their combined effects, we incubated slices in a cocktail of both inhibitors (100 μM MFA and 200 μM Ba^2+^) together for 1 h and examined neuronal responses (Figure 3, Supplementary Figure 2). Compared to control, MFA + Ba^2+^ condition exhibited a much smaller fold change in the peak fiber volley amplitude (aCSF: 1.1 [1.0–1.3], MFA + Ba^2+^: 0.2 [0.1–0.4] *p* < 0.0001; Figure 3B) as well as a decrease in the fEPSC amplitude (aCSF: 1.7 [1.4–1.9], MFA + Ba^2+^: 0.8 [0.3–1.3], *p* < 0.0001, Figure 3C; also see Supplementary Figures 2M, N). However, the fEPSC:fiber volley ratio after MFA + Ba^2+^ (0.4 [0.2–0.7]) was not different compared to controls (0.6 [0.5–0.6], *p* = 0.07; Figure 3D). We suggest this effect can be attributed mainly to the large decrease in fiber volley and indicates that the fEPSC decline was likely a result of fiber volley decline.

The fiber volley peak amplitude was so low in MFA + Ba^2+^ even in the first trace (Figure 3A) that time to 50% amplitude over the course of the 3 s, 20 Hz stimulation could not be accurately calculated. However, fEPSC amplitude often appeared in the first recording followed by a sharp decrease in amplitude (Figure 3A, left), which made it more feasible to calculate the time to 50% amplitude. The fEPSC amplitude decreased strongly, showing a decrease in fold change of time to 50% amplitude from 0.6 [0.5–1.0] before treatment to 0.1 [0.0–0.3] (*p* < 0.0001; Figures 3E, F; also see Supplementary Figure 2O) after 1 h in MFA + Ba^2+^ compared to aCSF control.

**FIGURE 4:**
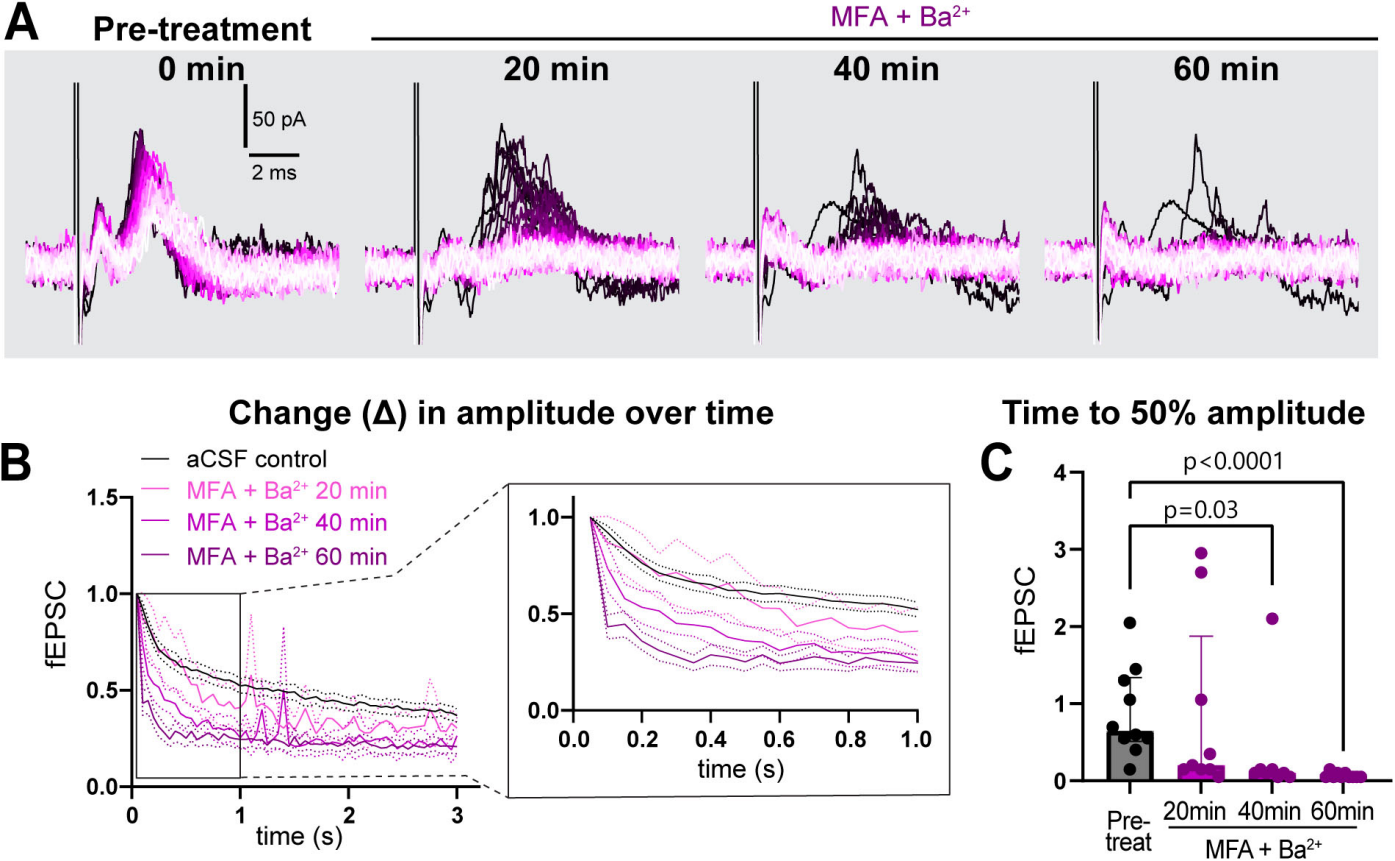
Simultaneous treatment with MFA and Ba^2+^ progressively worsened sustained neuronal activity. **(A)** Representative traces of response progression over 1 h in MFA + Ba^2+^-treated slices, shown at pre-treatment, 20, 40 and 60 min. **(B)** Average amplitude decrease of fEPSCs during 20 Hz stimulation in the pre-treatment (black, *n* = 10 slices), and in MFA + Ba^2+^ after 20 min (pink, *n* = 9 slices), 40 min (light purple, *n* = 8 slices) and 1 h (dark purple; *n* = 10 slices). Inset on the right shows an expanded view of the amplitude decrease during the first second. **(C)** Time to 50% fEPSC amplitude was decreased 40 and 60 min after co-applying MFA + Ba^2+^. aCSF control data are the same as in Figure 2. aCSF control *N* = 15 mice,15 slices; MFA + Ba^2+^ *N* = 8 mice, 8–10 slices. Kruskal-Wallis test; data shown as median [IQR].

This strong suppression of neuronal activity prompted us to record the response to stimulation train at in-between timepoints, 20 and 40 min (Figure 4A) in a subset of recordings. We found that time to 50% of fEPSC amplitude decreased progressively faster over the 1-h period (pre-treatment: 0.7 [0.5–1.3]; 20 min post-treatment: 0.2 [0.2–1.9], *p* > 0.9; 40 min: 0.1 [0.1–0.2], *p* = 0.03, 60 min: 0.1 [0.1–0.1], *p* < 0.0001, (Figures 4B, C). These data suggest that both K_*ir*_ and GJs coordinate to maintain low [K^+^]_*e*_ near nerve terminals and synapses to sustain neuronal excitability during long bouts of activity.

### 3.4 Genetic reduction of gap junctions has a mild effect on neuronal excitability

To further probe the role of astrocyte GJs on neuronal excitability, we used a genetic mouse model featuring a global knockout of Cx30 (Cx30KO) combined with an astrocyte-specific knockdown of Cx43 (Cx43KD), achieved by ICV injection of AAV9-GfaABC_1_D-Cre virus into Cx43*^fl/fl^* mice containing *^fl–stop^*tdTomato reporter (Supplementary Figure 5A). We refer to these mice as Cx30KO:Cx43KD. Viral targeting efficiency was measured by tdTomato-coverage in brain slices, which was calculated to be 96.2% [95.3–99.0] of total area (Supplementary Figures 5B, C). Despite highly efficient targeting of astrocytes by the virus, we quantified only a 40% reduction in Cx43 protein levels, as measured by western blotting (Supplementary Figures 5D, E). Immunolabelling for Cx30 showed a 90% reduction in Cx30 puncta compared to WT (*p* < 0.0001, Supplementary Figures 5F, G). However, littermate controls, which in our breeding scheme were heterozygous for Cx30, also showed a 67% reduction in Cx30 puncta (*p* = 0.01, Supplementary Figures 5F, G), indicating that they also had partial reduction of GJ coupling. For this reason, we compared data from Cx30KO:Cx43KD mice to wild-type (WT) controls (data from littermate controls are shown in Supplementary Figure 6).

**FIGURE 5:**
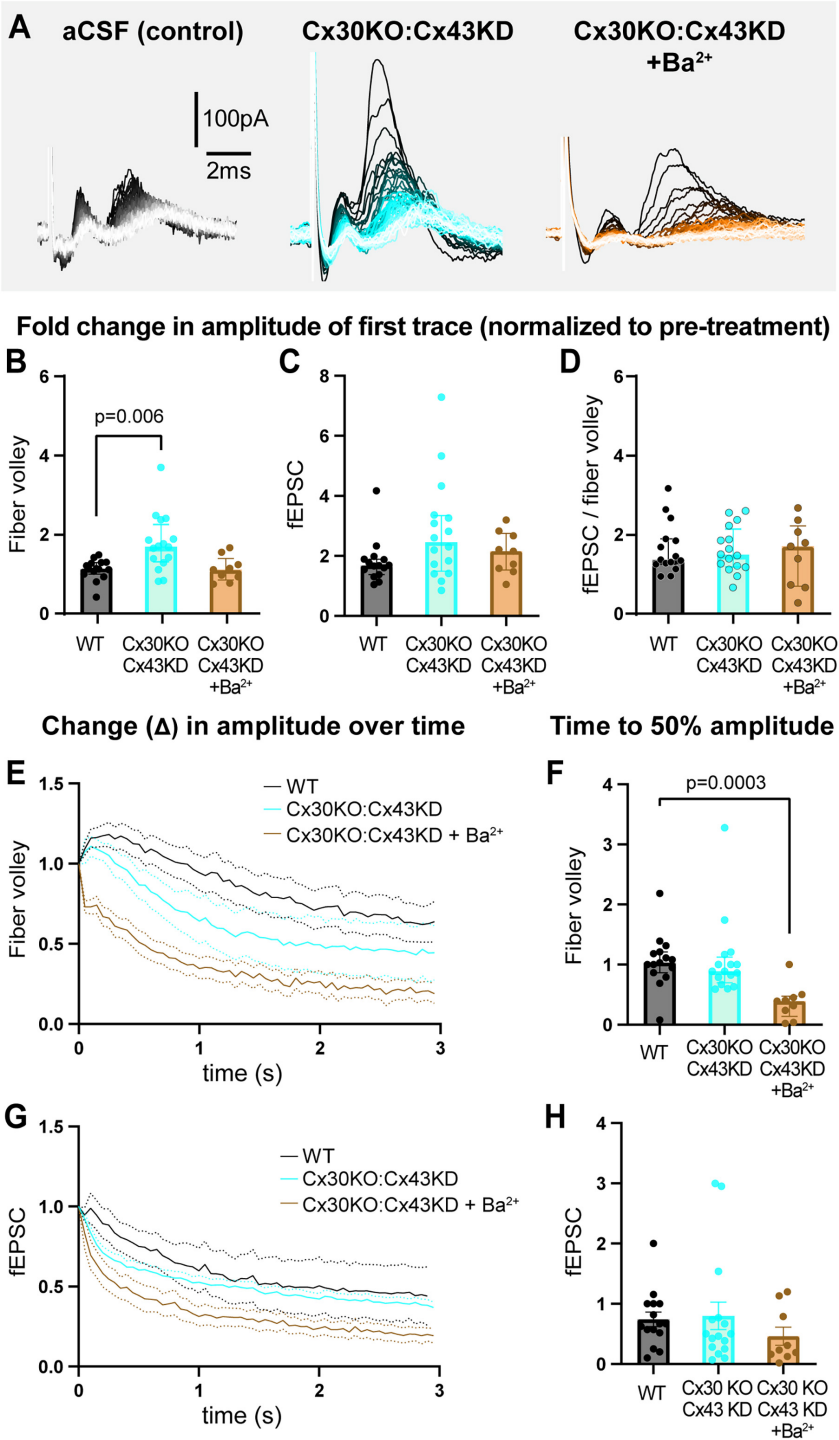
Genetically reducing GJs in astrocytes results in mild dysregulation of neuronal excitability. **(A)** Representative traces of recordings from slices from WT, Cx30KO:Cx43KD mice, and Cx30KO:Cx43KD + Ba^2+^. **(B)** The fold change in the amplitude of first fiber volley showing a relative increase in Cx30KO:Cx43KD, but not in Cx30KO:Cx43KD + Ba^2+^, compared to WTs. **(C)** The fold change in the amplitude of the first fEPSC was similar in all three conditions. **(D)** The fEPSC:fiber volley ratio showed no difference between groups. **(E,F)** The decrease in fiber volley amplitude over 3 s, 20 Hz stimulation after 1 h in each condition **(E)** showing a decrease in time to 50% amplitude in CX30KO:Cx43KD + Ba^2+^ only **(F)**. **(G,H)** The decrease in fEPSC amplitude over 3 s, 20 Hz stimulation after 1 h **(G)** showing no difference in time to 50% between groups **(H)**. WT control *N* = 15 mice, 15 slices; Cx30KO;Cx43KD *N* = 10 mice, 16 slices; Cx30KO:Cx43KD + Ba^2+^. *N* = 5 mice, 9 slices. Kruskal-Wallis test; data shown as median [IQR].

We repeated the 3 s, 20 Hz stimulation in slices from Cx30KO:Cx43KD without (GJ manipulation only) or with 200 μM Ba^2+^ (GJ manipulation + K_*ir*_ channel blocker; Figure 5A, Supplementary Figure 6). Compared to WT, the fold change in amplitude of the first fiber volley was 54% bigger in Cx30KO;Cx43KD condition (WT: 1.1 [1.0–1.3]; Cx30KO:Cx43KD: 1.7 [1.3–2.3], *p* = 0.006, Figure 5B), but not different in Cx30KO;Cx43KD + Ba^2+^ (1.1 [0.9–1.4], *p* > 0.9, Figure 5B). Surprisingly, Cx30KO:Cx43KD slices, without or with Ba^2+^, showed no difference in the fold change in peak fEPSC amplitude nor the fiber volley:fEPSC ratio (fEPSC, WT: 1.7 [1.4–1.9]; Cx30KO:Cx43KD: 2.5 [1.5–3.3], *p* = 0.07; Cx30KO:Cx43KD + Ba^2+^: 2.2 [1.5–2.8], *p* = 0.6; fiber volley:fEPSC ratio, WT: 1.4 [1.3–1.9]; Cx30KO:Cx43KD: 1.5 [1.2–2.2], *p* > 0.99; Cx30KO:Cx43KD + Ba^2+^ = 1.7 [0.7–2.2], *p* > 0.99; Figures 5C, D).

When we examined the decay in neuronal activity over the 3 s, 20 Hz stimulation, we observed that fold change in time to 50% amplitude of fiber volleys was comparable between Cx30KO:Cx43KD and WT (WT: 1.0 [0.9–1.2]; Cx30KO:Cx43KD: 0.9 [0.7–1.1], *p* > 0.9; Figures 5E, F), but decreased 60% faster when Ba^2+^ was also present, compared to WT controls (0.4 [0.1–0.5], *p* = 0.0003, Figures 5E, F). In contrast, fold change in time to 50% of fEPSCs was not statistically different between groups (WT: 0.7 [0.5–1.0]; Cx30KO:Cx43KD: 0.5 [0.3–0.8], *p* = 0.7; Cx30KO:Cx43KD + Ba^2+^: 0.2 [0.1–1.0], *p* = 0.2; Figures 5G, H; also see Supplementary Figures 6G–L). These data indicate that partial reduction of GJs can mildly alter neuronal excitability but is insufficient to robustly change it.

## 4 Discussion

Our goal was to evaluate the role of GJs on K^+^ buffering and thereby neuronal excitability. We measured the effect of acute block of GJs and K_*ir*_ channels (with MFA and Ba^2+^, respectively), as well as of genetic reduction of GJs, on field recordings of cortical neuronal activity. Our pharmacological data indicate that both GJs and K_*ir*_ channels modulate neuronal excitability in a similar manner, thus likely by contributing to K^+^ buffering. Furthermore, neuronal activity is bidirectionally sensitive to this modulation: GJs and K_*ir*_ channels can induce hyperexcitability of single evoked responses or sparse activity, as demonstrated by the increased amplitude of the first response, while suppressing sustained responses during prolonged activity, likely due to depolarization of the neuronal membrane (Larsen and MacAulay, 2014; Somjen, 2002).

Inhibiting both GJs and K_*ir*_ together by co-application of MFA and Ba^2+^ strongly disrupted neuronal responses to 3 s, 20 Hz stimulation. Importantly, we noticed that, when applied individually, each inhibitor took a long time (1 h) to exert robust disruption of neuronal activity, whereas applying both simultaneously had a robust and rapid effect within only 20–30 min. This was surprising, as we expected that once K_*ir*_ channels are blocked, a further block of GJs would not produce an additional effect, as they target the same pathway in buffering [K^+^]_*e*_. However, astrocytes are known to express a large number of both GJs and K_*ir*_4.1 channels that need to be sufficiently inhibited to observe this effect and co-application of drugs to simultaneously inhibit both mechanisms likely increased the efficiency with which K^+^ buffering is impaired, thus producing a faster and stronger disruption of neuronal excitability. Both drugs robustly block their respective targets, increasing confidence that these acute effects are due to GJs and K_*ir*_ channels. It has been reported that 100 μM MFA applied for 1 h blocks 99.3% of gap junctions (Veruki and Hartveit, 2009; Zhong et al., 2023), whereas 100 μM Ba^2+^ is sufficient to block 100% of K_*ir*_4.1 conductance on isolated Muller cells (Solessio et al., 2000) and astrocytes in slices (Zhou et al., 2021), with most studies in slices using it at 100–200 μM concentration range (Olsen et al., 2006; Procacci et al., 2023; Tyurikova et al., 2025).

Our genetic model aimed at reducing astrocyte GJs had complete global loss of Cx30 but only a partial (40%) reduction in Cx43 protein, even though the viral Cre hit almost all astrocytes as shown by tdTomato expression. Hence, we were successful in only partially decoupling the astrocyte syncytium. It is thus no surprise that Cx30KO:Cx43KD mice showed only a modest disruption of excitability. Previous studies have demonstrated that the coupling coefficient of astrocytes is very high at ∼94% (Ma et al., 2016), which suggests that the level of knockdown we were able to achieve may not be sufficient to robustly electrically decouple the syncytium (also see Section “4.3 K^+^ homeostasis: spatial or electrical buffering?”). Surprisingly, adding Ba^2+^ to Cx30KO:Cx43KD slices also produced a mild effect on neuronal excitability, affecting solely the amplitude decrease rate of the fiber volley during sustained activity. Overall, however, the similar directionality of the changes we observe in the partial GJ knockdown model is in agreement with data from acute MFA treatment experiments, where a full blockade of the GJs is predicted.

### 4.1 K^+^ accumulation: progression from hyperexcitability to suppression of activity

Previous work on K^+^ accumulation follows the pattern from hyperexcitability to suppression of neuronal activity; smaller K^+^ accumulation (6 mM) produces larger evoked somatosensory responses, whereas larger accumulation (12 mM) leads to strong suppression of responses, which were completely abolished at high K^+^ concentrations (17 mM) (Bazzigaluppi et al., 2017). This effect is analogous to our findings: partial decoupling of GJs (Cx30KO:Cx43KD) caused a mild increase in excitability, while a more robust block of GJs or K_*ir*_ channels (MFA or Ba^2+^, respectively) resulted in initial hyperexcitability, and complete block of K^+^ buffering system (MFA + Ba^2+^ together) strongly suppressed neuronal activity.

Indeed, increases in [K^+^]_*e*_ produce an initial facilitation of postsynaptic currents due to increased pre-synaptic Ca^2+^ influx and higher neurotransmitter release, but result in inactivation during prolonged stimulation (Augustine, 1990), likely due to neurotransmitter depletion as well as action potential failures and synaptic depression (Meeks and Mennerick, 2004) caused by inactivation of voltage-gated Na^+^ channels. We expect that applying each blocker individually lead to a moderate [K^+^]_*e*_ increase, showing an initially hyperexcitable response that could not be sustained, while simultaneous block of both GJs and K_*ir*_ lead to a higher [K^+^]_*e*_ increase and much stronger suppression of activity. This could explain why blocking GJs in epilepsy models, which have pronounced [K^+^]_*e*_ accumulation, results in suppression of hyperexcitability (Chever et al., 2016). On the other hand, blocking GJs alone under physiological conditions produces hyperexcitable responses (Wallraff et al., 2006), as we also observed in this study.

Given the importance of K^+^ homeostasis for brain health, functional redundancy must exist; [K^+^]_*e*_ can also be regulated by other mechanisms not involving K_*ir*_ and GJs, for example by Na^+^/K^+^-ATPase pumps on neurons and astrocytes (D’Ambrosio et al., 2002; Larsen and MacAulay, 2014; MacAulay, 2020), although its role in K^+^ buffering by astrocytes is still unclear. We observed stronger evoked activity in the first recording of each stimulation train, after an extended period of rest. This hyperexcitability was present in the first few responses even after short periods of recovery (i.e., the 2-min intervals between 3 trials for each condition), suggesting that other mechanisms must be sufficient to maintain [K^+^]_*e*_ within a physiological range given enough time. However, under sustained activity, as is the case during 3 s, 20 Hz stimulation, these systems appear unable to keep up. Notably, cortical neurons fire between 20 and 100 Hz frequencies, underscoring the relevance of the disruption we describe here (Tateno et al., 2004). The particularly strong effect of the acute co-treatment with MFA + Ba^2+^ indicates that K_*ir*_ and GJs together play a major role in maintaining physiological activity of cortical neurons, although data from the genetic model also indicate that other adaptive mechanisms can partially compensate for their function upon prolonged reduction in GJ coupling.

### 4.2 Gap junction toolbox: pharmacology and knockout models

Effective and specific block of astrocyte GJs remains a challenge in the field. Because GJs are present throughout the brain in many cell types, genetic knockout strategy would ideally be the best way to reduce GJ coupling specifically in astrocytes. However, genetic knockout models are also complicated by compensatory or adaptive responses to neuronal firing. This issue seems to be the case for astrocyte-specific K_*ir*_4.1 cKOs (using GFAP:Cre), which do not survive past 30 days but exhibit intact action potential firing at P15-P20 (Djukic et al., 2007). In contrast, mice with global Cx30 knockout and astrocyte-specific Cx43 knockout (also using GFAP:Cre) were reported to look healthy, yet show spontaneous seizures and evoked-hyperexcitability (Wallraff et al., 2006). A recent study used an inducible conditional astrocyte-specific knockdown of Cx30 and Cx43 (controlled by GLAST:Cre*^ERT2^*) featuring partial loss of GJs and showed that post-synaptic excitability was enhanced despite a decrease in pre-synaptic excitability (Hösli et al., 2022), in agreement with our observations using pharmacological blockers. However, they also showed that partial KD of astrocyte GJs induced a reactive astrocyte phenotype and resulted in wide-ranging alterations in the proteins present in them (Hösli et al., 2022), making it difficult to attribute the changes in neuronal excitability to GJs alone. It is possible that our genetic model had similar compensatory/adaptive changes in astrocytes (as well as neurons and other glia), which contributed to the milder effects we observed upon genetic manipulation of GJs even when co-treated with Ba^2+^.

Acute inhibition with pharmacological blockers circumvents compensatory genetic changes and allows for more robust block of GJs, but are, in turn, limited by non-cell-specific action of the inhibitors. One limitation of our pharmacological study is the use of 200 μM Ba^2+^, which robustly blocks K_*ir*_ channels, but also can block K_*ATP*_ channels and large-conductance K^+^ (BK) channels at this concentration (Srivastava et al., 2024; Takano and Ashcroft, 1996), both of which are present on cortical neurons. K_*ATP*_ channels couple neuronal excitability and metabolism and are thought to reduce hyperexcitability in pathophysiology. Under physiological conditions, they are mostly closed (Lv et al., 2022) but can occasionally be activated (Tanner et al., 2011). Thus, it is possible that K_*ATP*_ channels are minimally contributing to the effect we report, especially the faster decay during sustained activity. The effect of Ba^2+^ on BK channels (Srivastava et al., 2024; Zhou et al., 2012) could also potentially contribute to the effect we observe, however, previous work has shown that BK channels contribute minimally to pre- and post-synaptic activity in cortical neurons even during burst activity in response to a continuously depolarizing stimulus (Bock and Stuart, 2016). Therefore, although we cannot exclude BK channel-mediated effects from our study, we believe they are unlikely. The concentration of GJ blocker MFA we employed (100 μM) has been shown to effectively block astrocyte GJ coupling (Ma et al., 2016), although it also has some non-specific effects, including activation of KNCQ (Peretz et al., 2005) channels and inhibition of TRP channels (Klose et al., 2011; Kövesdi et al., 2025) which can suppress activity (Peretz et al., 2005; Riquelme et al., 2021). These effects are unlikely to be involved in the early hyperexcitability observed in our preparation, but we cannot exclude that they contribute, at least in part, to the suppression during sustained activity. MFA would also block the GJs on inhibitory neurons, but these have been demonstrated not to directly regulate overall excitability (Curti et al., 2022).

Both pharmacological and genetic strategies have considerable benefits and limitations. That blocking GJ or K_*ir*_ channels acutely produced a similar effect indicates that they both contribute to the K^+^ buffering system, and that genetically induced partial decoupling produces a similar trend supports this overall concept. Importantly, our data showed that the variability in the astrocyte GJ literature might be driven by the differences in stimulation paradigms or approaches used for GJ reduction. Developing more precise and effective cell-specific tools and strategies, such as astrocyte-specific blockers or effective (and complete) adult knockdown of GJs, will be crucial for further elucidating the role of astrocyte GJs and K_*ir*_4.1 in regulating K^+^ homeostasis.

### 4.3 K^+^ homeostasis: spatial or electrical buffering?

The interaction between K_*ir*_ and GJs largely explains passive electrical properties of mature astrocytes and is necessary for K^+^ buffering, as shown by experimental data and computational modeling (Ma et al., 2016; Zhou et al., 2021). Astrocytes are an ideal candidate for buffering K^+^ because of two key features set by K_*ir*_ channels and GJs: (1) K_*ir*_4.1 (alongside other K^+^ channels) sets the resting membrane potential of astrocytes, hyperpolarizing them by bringing the membrane potential values close to E_*k*_. As a result, during neuronal firing, astrocytes are highly sensitive to [K^+^]_*e*_ increases, which is why they depolarize (albeit to a much smaller degree) in synchrony with neurons (De Saint Jan and Westbrook, 2005); (2) coupling of astrocytes via GJs provides an “electrical buffering” system and equilibrates the membrane potential (Zhong et al., 2023), keeping depolarizations to a minimum in the face of large increases of K^+^ uptake. Computational modeling indicates that prolonged depolarization of astrocyte membrane introduces instability that would affect proper astrocyte functions (Janjic et al., 2023). The role of GJs in allowing currents to leak between coupled cells, thus stabilizing resting membrane potential and dampening excitability, is a property that is well documented for electrical synapses among interneurons (Alcami and Pereda, 2019). Importantly, in astrocytes, GJs help maintain their relatively hyperpolarized membrane potential, which sustains the driving force for continuous K^+^ removal from the extracellular space. Without GJs, K_*ir*_-mediated K^+^ entry would produce large depolarizations of astrocytes and impair their capacity to take up K^+^ during neuronal firing (Ma et al., 2016; Pannasch et al., 2012). This framework suggests that GJs contribute to K^+^ buffering not as much by physically moving K^+^ between astrocytes (spatial buffering) but rather by maintaining their membrane potential hyperpolarized to sustain the driving force for K^+^ uptake (electrical buffering). Thus, perhaps our theoretical framework should be revised to think of the astrocyte syncytium less as a spatial buffering system and more as an electrical buffering system.

Our observation that K_*ir*_ and GJs contribute similarly to neuronal excitability and strongly impair neuronal activity when blocked together aligns with this framework; larger astrocyte depolarizations would lead to stronger disruption of [K^+^]_*e*_ homeostasis and, therefore, of neuronal activity. Existing discussions of astrocyte-mediated K^+^ buffering often do not appropriately account for the electrical coupling bestowed by GJs and thus assume that K^+^ entry-mediated depolarizations are bigger (Larsen and MacAulay, 2014). Consequently, they underestimate the buffering capacity of astrocytes. In contrast, accounting for K_*ir*_ and GJs in the way we propose is in line with the idea of the development of electrical buffering system and the electrically passive feature of the astrocyte syncytium (Zhong et al., 2023; Zhou et al., 2021). Indeed, these passive properties may be exactly what allows astrocytes to have an active and continuous role in regulating neuronal excitability.

## 5 Conclusion

In summary, we suggest that GJs and K_*ir*_ channels bidirectionally and jointly regulate neuronal activity, restraining hyperexcitability during sparse firing conditions and preventing neuronal fatigue during sustained activity. We propose that this is largely due to the passive properties endowed on astrocytes by GJs, thus preserving a constant drive for K^+^ influx through K_*ir*_ channels and hence maintaining [K^+^]_*e*_ within physiological bounds to sustain neuronal activity. Astrocytes appear to maintain this role even when their GJs are partially reduced. Better understanding how disruptions of passive properties due to changes in K_*ir*_ channels or GJs on astrocytes affect neuronal circuits could provide valuable insights into brain disorders with disrupted neuronal excitability, such as epilepsy (Kinboshi et al., 2020), ischemia (Xu et al., 2010), and autism (Davoudi et al., 2025).

## Data Availability

The raw data supporting the conclusions of this article will be made available by the authors, without undue reservation.

## Supplementary material

Supplementary Figure 1 Response stabilization over 30 min. **(A)** Representative traces showing the increase in field response amplitude over 30 min. **(B)** Both fiber volley (left) and fEPSC amplitude (right) increased over 30 min of stabilization. *N* = 9 mice, 10 slices. Wilcoxon test.

Supplementary Figure 2 Effect of GJs and K_*ir*_ channels on neuronal field responses. **(A–C)** Amplitude of the first fiber volley did not change after 1 h in aCSF **(A)** or Ba^2+^ **(C)**, but decreased in MFA **(B)**. **(D–F)** Amplitude of the first fEPSC increased after 1 h in all three conditions. **(G–I)** Change in normalized fiber volley amplitude over the 3 s, 20 Hz stimulation (left) and time to 50% of maximum decrease (right) showed no difference between pre-treatment and after 1 h in aCSF **(G)**, MFA **(H)**, or Ba^2+^ **(I)**. **(J–L)** Change in fEPSC amplitude over the 3 s, 20 Hz stimulation (left) and time to 50% of maximum decrease (right) shows a decrease after 1 h in aCSF **(J)**, MFA **(K)**, and Ba^2+^ **(L)** compared to pre-treatment. **(M,N)** The amplitude of first fiber volley decreased after 1 h in MFA + Ba^2+^ **(M)**, but that of fEPSC did not **(N)**. **(O)** Change in fEPSC amplitude over the 3 s, 20 Hz stimulation (left) and time to 50% of maximum decrease (right) shows a strong decrease after 1 h in MFA + Ba^2+^. aCSF control *N* = 15 mice, 15 slices; MFA *N* = 13 mice, 13 slices, Ba^2+^ *N* = 13 mice, 14 slices, MFA + Ba^2+^ *N* = 11 mice, 16 slices. Wilcoxon test.

Supplementary Figure 3 Peak amplitude variability is not explained by age or sex. **(A)** There was no correlation between mouse age and peak amplitude of either fiber volley or fEPSC (*n* = 28 mice, 53 slices, linear correlation). **(B)** There was no difference in peak amplitude of either fiber volley or fEPSC between males and females. *N* = 14 female, 24 slices; *N* = 14 male, 29 slices; Mann-Whitney test.

Supplementary Figure 4 Carbenoxolone (CBX) had a mild effect on neuronal responses. **(A,B)** Fiber volley amplitude **(A)** was increased after 1 h in CBX while fEPSC amplitude **(B)** was unchanged (Wilcoxon test). **(C–E)** The fold change in the amplitude of fiber volley **(C)** and fEPSC **(D)**, compared to pre-treatment values, as well as the fEPSC:fiber volley ratio **(E)** were unchanged in CBX compared to aCSF control after 1 h (Mann Whitney test). **(F,G)** Amplitude decrease of fiber volley over the 3 s, 20 Hz stimulation **(F)** showed a slightly faster time to 50% **(G)** after 1 h in CBX (Wilcoxon test). **(H,I)** Amplitude decrease of the fEPSC over 3 s, 20 Hz stimulation **(H)** and time to 50% **(I)** showed no difference after 1 h in CBX (Wilcoxon test). aCSF *N* = 15 mice, 15 slices; CBX *N* = 8 mice, 8 slices. Data shown as median [IQR].

Supplementary Figure 5 Genetic GJ reduced mouse model. **(A)** Overview of mouse model for global Cx30 KO and astrocyte-targeted Cx43 KD as well as littermate controls. **(B,C)** Representative image of tdTomato **(B)** showing near-complete astrocyte targeting by the AAV9-GfaABC_1_D virus in Cx30 KO:Cx43 KD model (**C**, *N* = 3 mice, 13 slices). **(D,E)** Western blot **(C)** analysis showed ∼40% reduction of Cx43 protein **(D)** in Cx30 KO:Cx43 KD model (*N* = 5 mice) compared to littermate controls (*N* = 4 mice, Mann-Whitney test). **(F)** Immunolabeling for Cx30 in wild-type, Cx30^+/–^ littermate, and Cx30^–/–^ mice. **(G)** Quantification of Cx30 puncta density shows a graded reduction of Cx30 protein in Cx30^+/–^ and Cx30^–/–^ mice WT: *N* = 3 mice, 5 sections; Cx30^+/–^: *N* = 6 mice, 12 slices, Cx30^–/–^: *N* = 10 mice, 13 slices. Mann-Whitney one-tailed test; data shown as median [IQR].

Supplementary Figure 6 Effect of genetically reducing astrocyte GJs on neuronal excitability. **(A–C)** Amplitude of the first fiber volley increased after 1 h in littermate control **(A)** and Cx30KO:Cx43KD **(B)**, not in Cx30KO:Cx43KD+Ba^2+^ **(C)**. **(D–F)** Amplitude of the first fEPSC increased after 1 h in all three conditions. **(G–I)** Change in fiber volley amplitude over the 3 s, 20 Hz stimulation (left) and time to 50% of maximum decrease (right) showing no difference between pre-treatment (0 min) and after 1 h in littermate controls **(G)** and Cx30KO:Cx43KD **(H)**, but a decrease in Cx30KO:Cx43KD+Ba^2+^ condition **(I)**. **(J–L)** Change in fEPSC amplitude over the 3 s, 20 Hz stimulation (left) and time to 50% of maximum decrease (right) shows a decrease after 1 h in all three conditions compared to pre-treatment. WT control *N* = 15 mice, 15 slices; Cx30KO;Cx43KD *N* = 10 mice, 16 slices; Cx30KO;Cx43KD+Ba^2+^ *N* = 5 mice, 9 slices. Wilcoxon test.
