# Supplementary figures and images for: Astrocyte gap junctions and Kir channels contribute to K+ buffering and regulate neuronal excitability
Source: Front Cell Neurosci. 2025 Nov 20;19:1571218. doi: 10.3389/fncel.2025.1571218 (PMC12676906; doi:10.3389/fncel.2025.1571218)

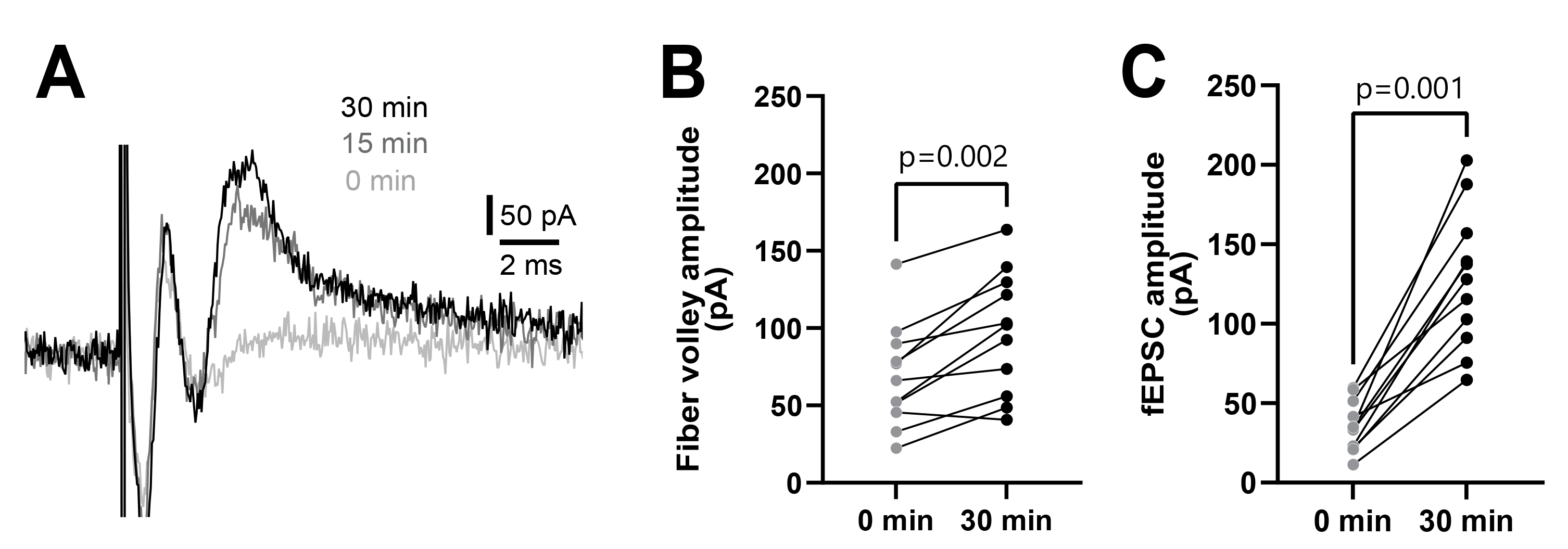

Supplement: Supplementary Figure 1 — Response stabilization over 30 min. (A) Representative traces showing the increase in field response amplitude over 30 min. (B) Both fiber volley (left) and fEPSC amplitude (right) increased over 30 min of stabilization. N = 9 mice, 10 slices. Wilcoxon test. [file Image_1.tif]

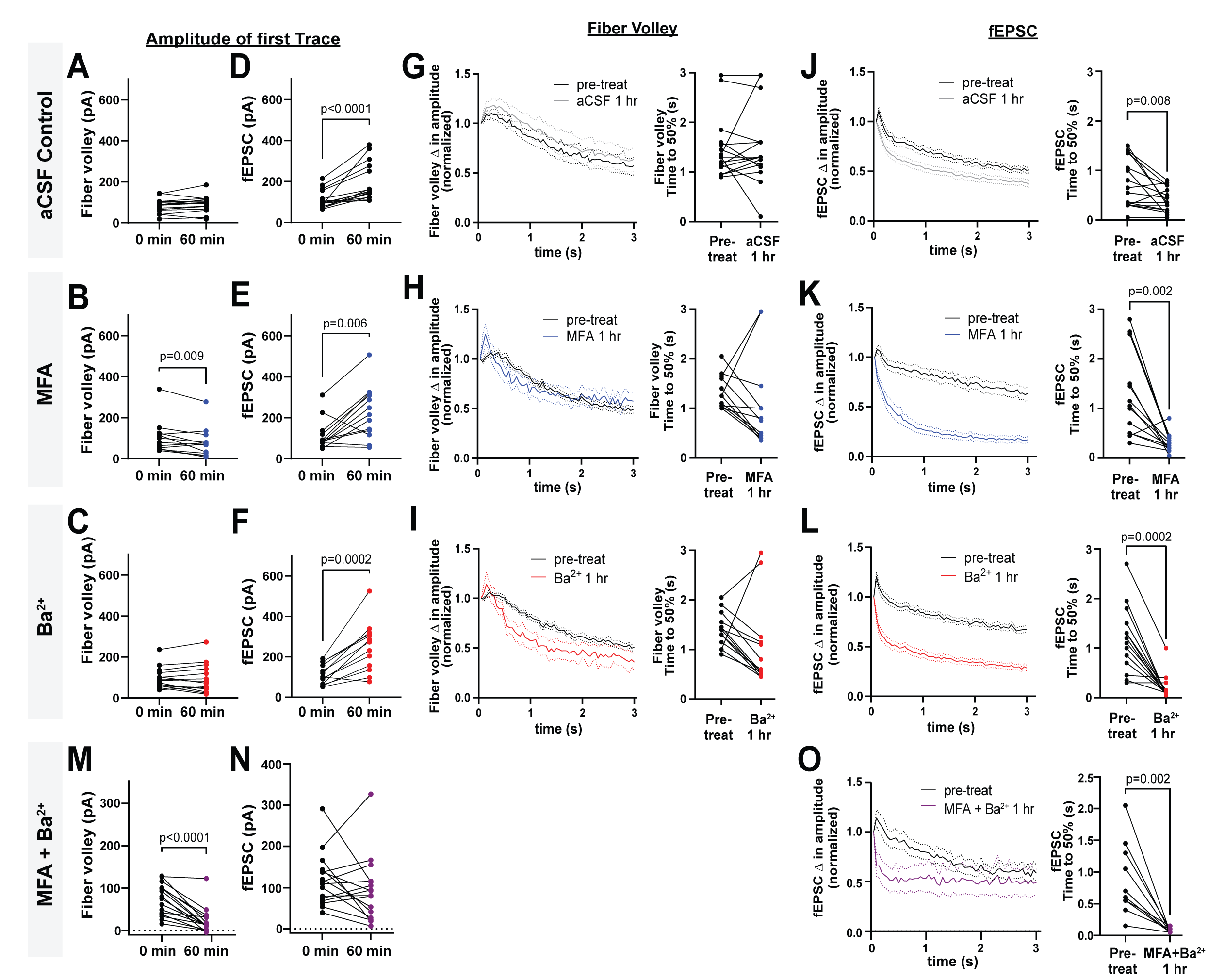

Supplement: Supplementary Figure 2 — Effect of GJs and Kir channels on neuronal field responses. (A–C) Amplitude of the first fiber volley did not change after 1 h in aCSF (A) or Ba2+ (C), but decreased in MFA (B). (D–F) Amplitude of the first fEPSC increased after 1 h in all three conditions. (G–I) Change in normalized fiber volley amplitude over the 3 s, 20 Hz stimulation (left) and time to 50% of maximum decrease (right) showed no difference between pre-treatment and after 1 h in aCSF (G), MFA (H), or Ba2+ (I). (J–L) Change in fEPSC amplitude over the 3 s, 20 Hz stimulation (left) and time to 50% of maximum decrease (right) shows a decrease after 1 h in aCSF (J), MFA (K), and Ba2+ (L) compared to pre-treatment. (M,N) The amplitude of first fiber volley decreased after 1 h in MFA + Ba2+ (M), but that of fEPSC did not (N). (O) Change in fEPSC amplitude over the 3 s, 20 Hz stimulation (left) and time to 50% of maximum decrease (right) shows a strong decrease after 1 h in MFA + Ba2+. aCSF control N = 15 mice, 15 slices; MFA N = 13 mice, 13 slices, Ba2+ N = 13 mice, 14 slices, MFA + Ba2+ N = 11 mice, 16 slices. Wilcoxon test. [file Image_2.tif]

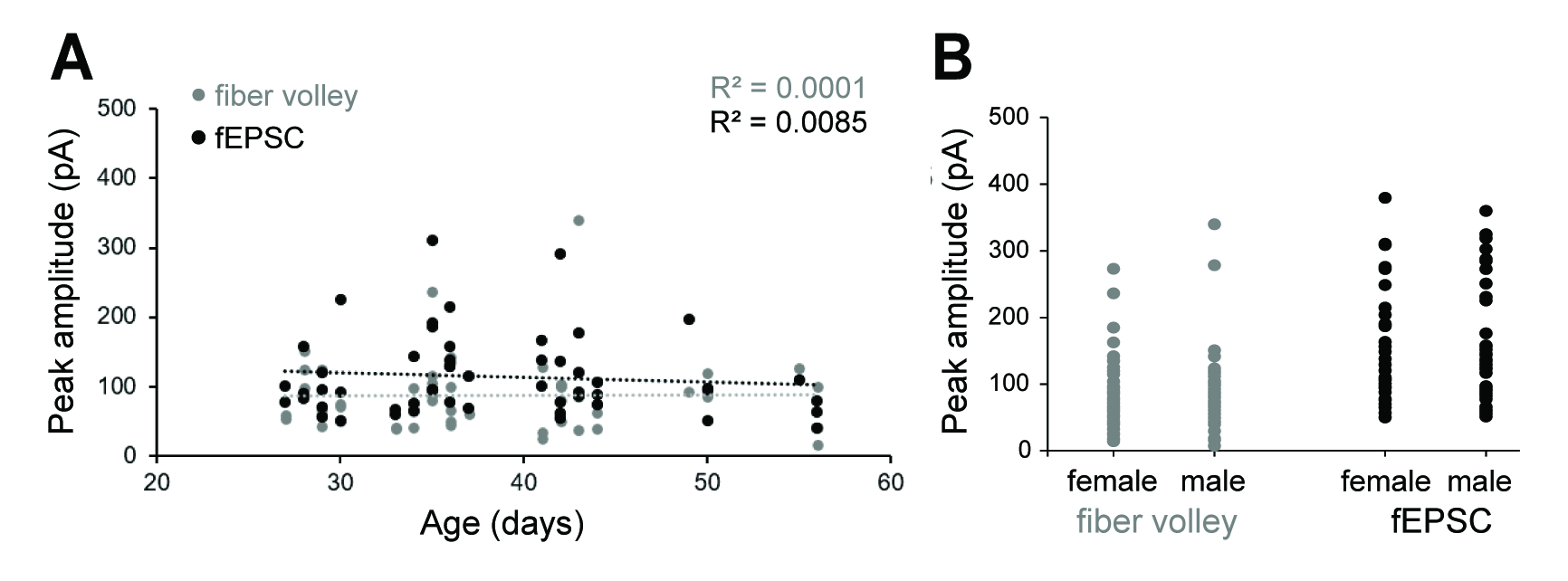

Supplement: Supplementary Figure 3 — Peak amplitude variability is not explained by age or sex. (A) There was no correlation between mouse age and peak amplitude of either fiber volley or fEPSC (n = 28 mice, 53 slices, linear correlation). (B) There was no difference in peak amplitude of either fiber volley or fEPSC between males and females. N = 14 female, 24 slices; N = 14 male, 29 slices; Mann-Whitney test. [file Image_3.tif]

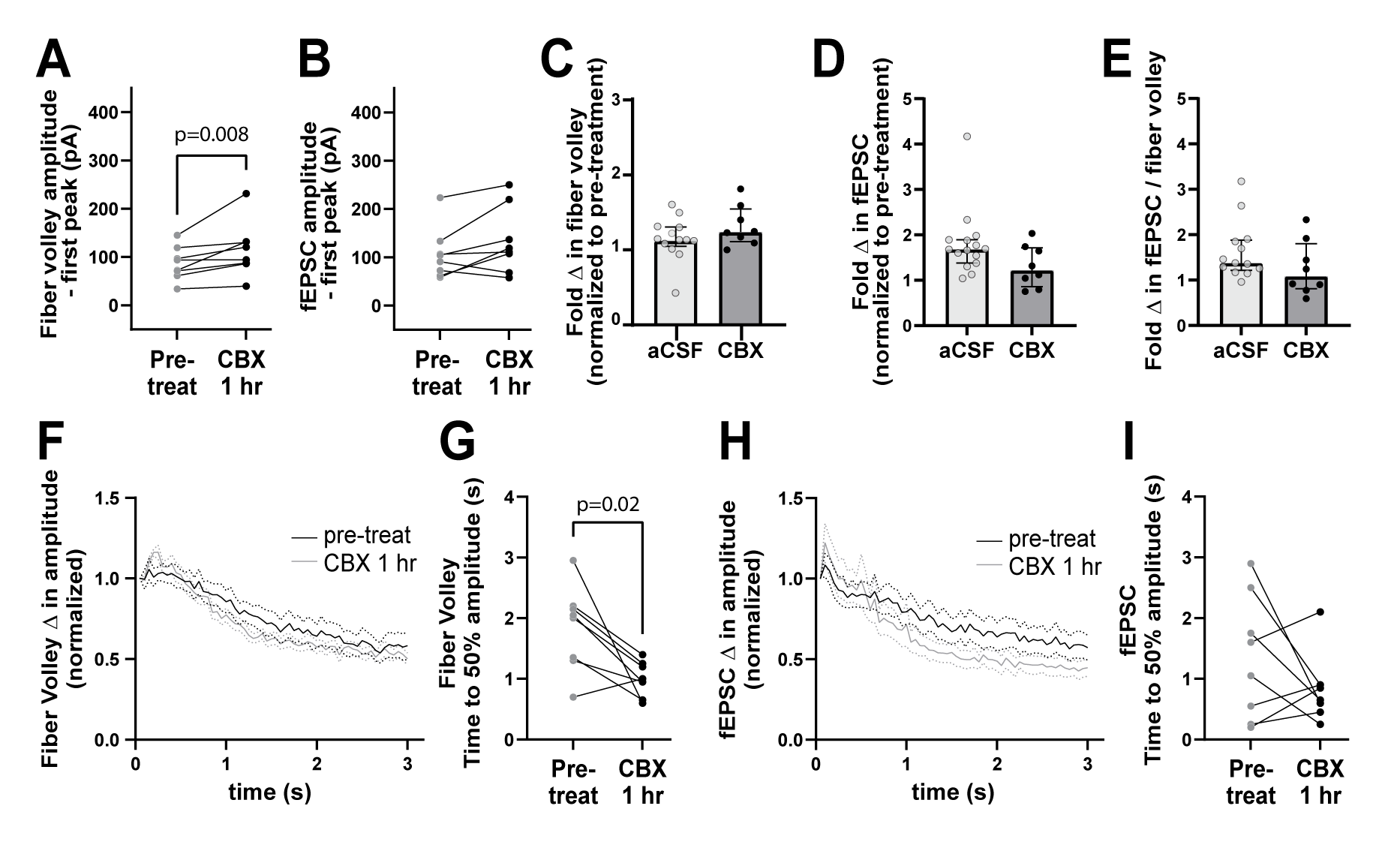

Supplement: Supplementary Figure 4 — Carbenoxolone (CBX) had a mild effect on neuronal responses. (A,B) Fiber volley amplitude (A) was increased after 1 h in CBX while fEPSC amplitude (B) was unchanged (Wilcoxon test). (C–E) The fold change in the amplitude of fiber volley (C) and fEPSC (D), compared to pre-treatment values, as well as the fEPSC:fiber volley ratio (E) were unchanged in CBX compared to aCSF control after 1 h (Mann Whitney test). (F,G) Amplitude decrease of fiber volley over the 3 s, 20 Hz stimulation (F) showed a slightly faster time to 50% (G) after 1 h in CBX (Wilcoxon test). (H,I) Amplitude decrease of the fEPSC over 3 s, 20 Hz stimulation (H) and time to 50% (I) showed no difference after 1 h in CBX (Wilcoxon test). aCSF N = 15 mice, 15 slices; CBX N = 8 mice, 8 slices. Data shown as median [IQR]. [file Image_4.tif]

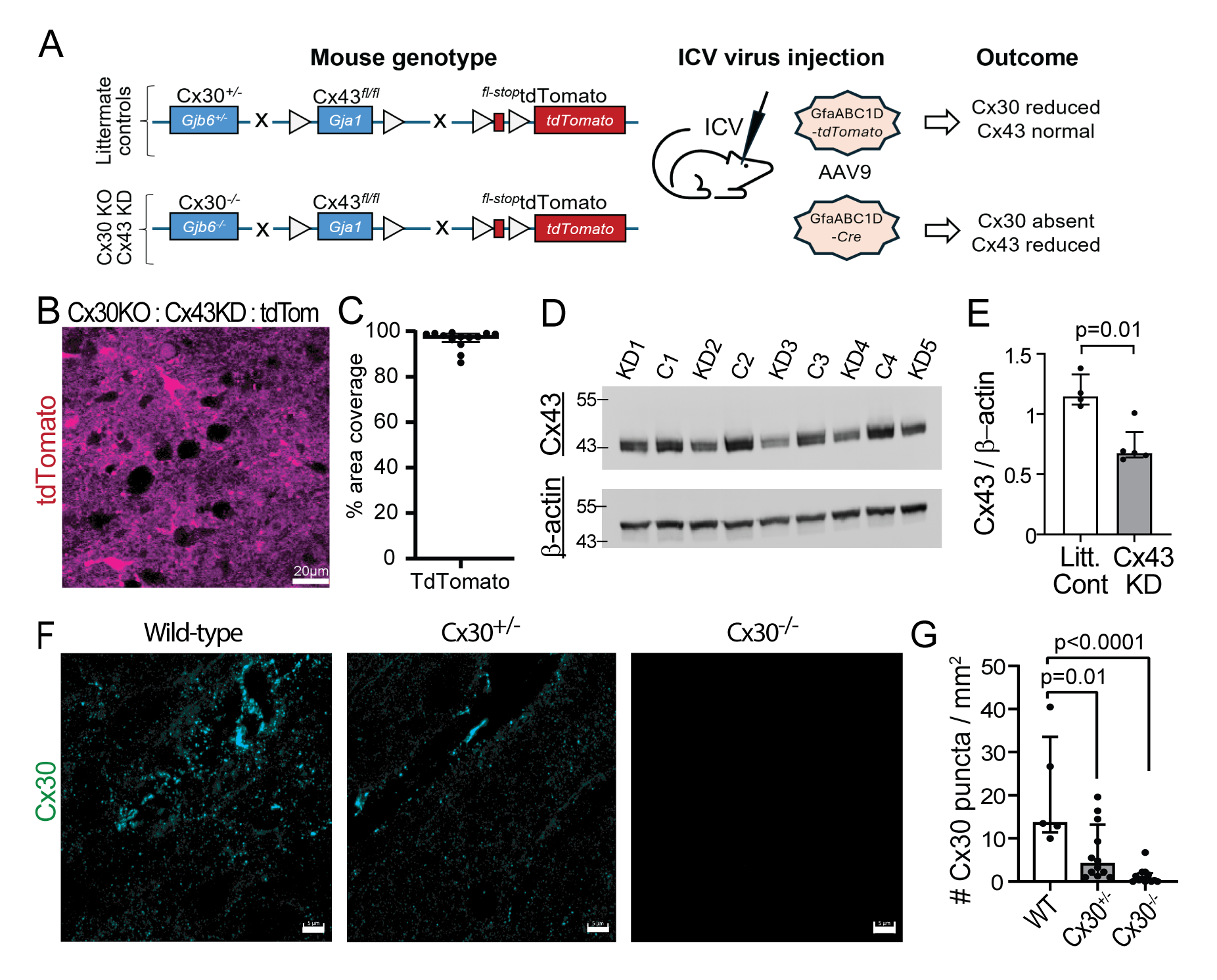

Supplement: Supplementary Figure 5 — Genetic GJ reduced mouse model. (A) Overview of mouse model for global Cx30 KO and astrocyte-targeted Cx43 KD as well as littermate controls. (B,C) Representative image of tdTomato (B) showing near-complete astrocyte targeting by the AAV9-GfaABC1D virus in Cx30 KO:Cx43 KD model (C, N = 3 mice, 13 slices). (D,E) Western blot (C) analysis showed ∼40% reduction of Cx43 protein (D) in Cx30 KO:Cx43 KD model (N = 5 mice) compared to littermate controls (N = 4 mice, Mann-Whitney test). (F) Immunolabeling for Cx30 in wild-type, Cx30+/– littermate, and Cx30–/– mice. (G) Quantification of Cx30 puncta density shows a graded reduction of Cx30 protein in Cx30+/– and Cx30–/– mice WT: N = 3 mice, 5 sections; Cx30+/–: N = 6 mice, 12 slices, Cx30–/–: N = 10 mice, 13 slices. Mann-Whitney one-tailed test; data shown as median [IQR]. [file Image_5.tif]

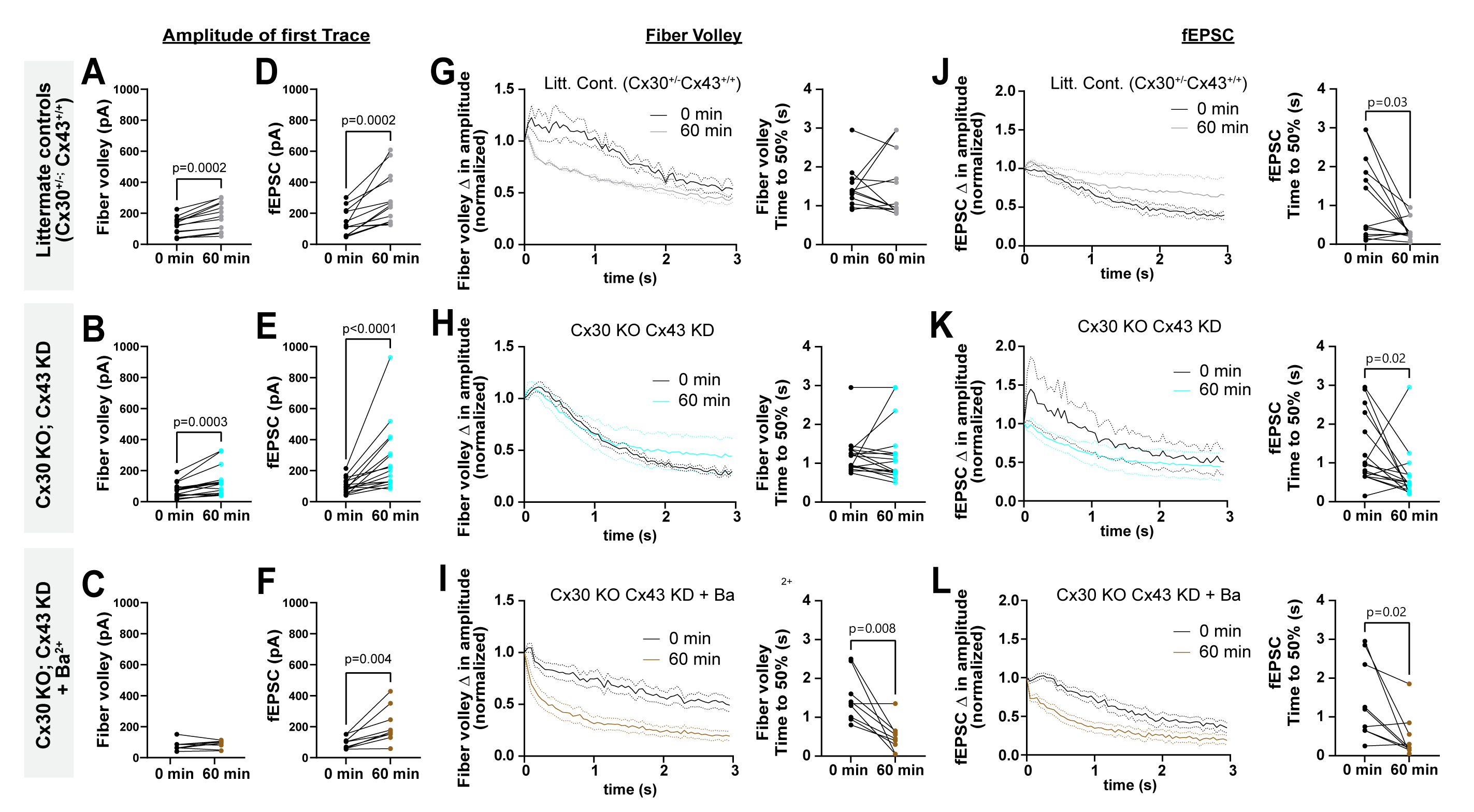

Supplement: Supplementary Figure 6 — Effect of genetically reducing astrocyte GJs on neuronal excitability. (A–C) Amplitude of the first fiber volley increased after 1 h in littermate control (A) and Cx30KO:Cx43KD (B), not in Cx30KO:Cx43KD+Ba2+ (C). (D–F) Amplitude of the first fEPSC increased after 1 h in all three conditions. (G–I) Change in fiber volley amplitude over the 3 s, 20 Hz stimulation (left) and time to 50% of maximum decrease (right) showing no difference between pre-treatment (0 min) and after 1 h in littermate controls (G) and Cx30KO:Cx43KD (H), but a decrease in Cx30KO:Cx43KD+Ba2+ condition (I). (J–L) Change in fEPSC amplitude over the 3 s, 20 Hz stimulation (left) and time to 50% of maximum decrease (right) shows a decrease after 1 h in all three conditions compared to pre-treatment. WT control N = 15 mice, 15 slices; Cx30KO;Cx43KD N = 10 mice, 16 slices; Cx30KO;Cx43KD+Ba2+ N = 5 mice, 9 slices. Wilcoxon test. [file Image_6.tif]
